# Exploring how ‘wish-granting’ interventions foster wellbeing for children with life-threatening health conditions and their families: A qualitative study

**DOI:** 10.1177/13674935241287865

**Published:** 2024-09-27

**Authors:** Gemma Heath, Cassandra Screti, Rebecca Knibb

**Affiliations:** School of Psychology, 1722 Aston University, Birmingham, UK

**Keywords:** Parents, child, chronic disease, psychology, positive, interview

## Abstract

Wish-granting is a form of positive psychological intervention that seeks to promote child wellbeing by fulfilling a wish of their choice. This study aimed to explore families’ experiences of receiving wish-granting interventions to understand how wishes impact wellbeing. Fifty in-depth semi-structured interviews were carried out with 22 families (23 parents, 17 young people); seven charity volunteers; and five health professionals, recruited from the United Kingdom. Interviews were transcribed verbatim and analysed using a thematic framework approach. Findings suggest wishes improve wellbeing by increasing positive emotion; by broadening families’ horizons; by providing an alternative focus; and by fostering opportunities for togetherness. To grow and maintain impact, consideration should be given to developing strategies that increase anticipation; keep wish memories alive; encourage children to make wishes that stretch their perceived limitations; and facilitate families to share their experiences and ‘give back’ to the community.

## Background

Life-limiting health conditions such as cancer and cystic fibrosis are those for which there is no cure or where curative treatment may be possible but not always successful (Together for Short Lives, 2018). In the United Kingdom, there are more than 86,000 children living with complex and life-threatening conditions (Fraser et al., 2020). While advances in treatment and technology means that these children are surviving for longer, doing so often involves living with physical symptoms, including pain, nausea, and fatigue, combined with intensive, complicated, and sometimes painful treatments, and periods of frequent hospitalisation (Barlow and Ellard, 2006). It is no surprise that living with a life-threatening health condition in childhood significantly disrupts life and wellbeing (Price et al., 2022; Smith et al., 2015).

Research shows that parents of children with complex conditions are also vulnerable to psychological distress (Fraser et al., 2021; Kaugars et al., 2018; Rosenberg et al., 2013) leading to poorer quality of life (Hatzmann et al., 2008). Siblings of children with life-threatening health conditions are also at significant risk of poorer mental health, with increased anxiety and depression and post-traumatic stress disorder, lower quality of life, and disruption to academic and social functioning (Alderfer et al., 2010; Limbers and Skipper, 2014; Vermaes et al., 2012). Living with a life-threatening health condition in childhood thus has a profound effect not only children themselves but also on their families (Price et al., 2022).

Interventions are needed to ensure psychological wellbeing for all family members when a child is living with a life-threatening health condition (Eccleston et al., 2019). Recognising health as more than the absence of illness (WHO, 2006) and wellbeing as a combination of feeling good and functioning well (WHO, 2004), interventions rooted in positive psychology aim not only to reduce distress but also to promote positive emotions and inner strengths, by supporting individuals to harness adaptive coping responses and positive adjustment (Ghosh and Deb, 2017). One form of positive psychological intervention provided to children with life-threatening health conditions is granting children’s wishes (Chaves et al., 2016). Often carried out by charitable organisations, wish-granting aims to promote child wellbeing by fulfilling a wish of their choice (Heath et al., 2021). Examples can include meeting a celebrity, going on a special holiday, receiving an important piece of equipment, and or experiencing a day in the life of a particular role or person (e.g. a train driver or a zookeeper).

A systematic review (Heath et al., 2021) shows that wish-granting interventions provide enjoyable experiences for children and their families, alongside positive impacts on child health behaviour (e.g. engagement with treatment), physical health outcomes (e.g. reduction in pain), and improvements to psychological wellbeing. A small but emerging evidence base indicates improvements in these domains for children themselves (Chaves et al., 2016; Ewing, 2007; Shoshani, 2016) and for wish-child parents (Darlington et al., 2013; Schilling and Sarigiani, 2014). Post-wish improvements have quantitatively been reported to include a reduction in nausea (Chaves et al., 2016), increase in energy levels (Darlington et al., 2013), improved physical strength (Schilling and Sarigiani, 2014), and improved functional skills (Chaves et al., 2016; Schilling and Sarigiani, 2014). Improvements to mental health and wellbeing have also been reported, including increased positive emotion and decreased symptoms of depression, sadness, anxiety, and fear (Chaves et al., 2016; Darlington et al., 2013; Schilling and Sarigiani, 2014; Shoshani et al., 2016).

While research has started to explore wish-granting interventions and child health, there remain gaps in our understanding (Heath et al., 2021). Heath et al.’s (2021) review found that of 10 included studies, most focused exclusively on children with cancer, thus neglecting other conditions eligible for wishes, no studies to date had been conducted in the United Kingdom; only three studies reported children’s perspectives; and only one provided a qualitative exploration of wish fulfilment. No studies have examined *how* wishes generate their impact and how that impact could be improved.

## Aim

The aim of this study was to explore UK families’ experiences of receiving wish-granting interventions to understand how wishes impact wellbeing.

## Methods

### Study design

A qualitative design employing semi-structured interviews within an interpretative framework (Crotty, 1998) was used, to capture of families’ views and experiences of receiving a wish from a UK-based, international wish-granting charity, as well as those of health professionals with experience of referring children for wishes and wish charity volunteers.

### Sample

To ensure different aspects of wish making and granting were captured, children, young people, and their parents were purposefully sampled from three categories: families of children who had made a wish (and received confirmation that they would receive their wish), but not yet had it granted (pre-wish); families of children who had their wish granted within 8 weeks of the interview (post-wish ≤8 weeks); and families of children who had their wish granted 8 weeks or longer before the interview (post-wish ≥8 weeks). Variation was also sought in child age, health condition, and wish type: ‘To go’ (e.g. on holiday), ‘To have’ (e.g. a computer), ‘To meet’ (e.g. a celebrity), and ‘To be’ (e.g. a fireman) (Heath et al., 2021). Health professionals and charity volunteers were sampled to capture multiple perspectives of wish granting.

### Recruitment

Eligible participants were identified by a UK-based wish-granting charity and sent a study information pack on behalf of the research team, including an invitation to participate and age-appropriate information sheets. Participants were invited to contact researchers directly to express interest in taking part. Once eligibility was established and participation agreed, written consent was obtained from parents (and assent from children) before an interview was arranged.

### Ethics

Ethical approval was provided by the Aston University Life and Health Sciences Ethics Review Committee on 19/1/2018 (#1281).

### Data collection

In-depth, semi-structured interviews were carried out by first and second authors. Interviews were conducted face-to-face, via telephone or via video call in accordance with participant preference. Face-to-face interviews took place in families’ homes or in an appropriate space (e.g. University room and quiet coffee shop). Family members were able to be interviewed together or separately. Interviews lasted just over an hour and were audio recorded. Arts-based methods (e.g. drawings, collages, and timelines) were used alongside interviews to support data collection. Interview questions were designed to elicit information on participant’s experiences of making a wish, including choosing a wish, waiting for a wish, expectations for wish fulfilment, wish experience, feelings following a wish, wish meanings, wish-child physical or emotional wellbeing, and ideas for wish improvement. Questions for health professionals focused on their experiences of referring children for a wish and observations of families following a wish. Volunteers were asked about their experiences of supporting families while making wishes and having them granted. Demographic information was collected from participants to support data analysis.

### Data analysis

Interviews were transcribed, anonymised, and analysed using Framework Analysis (Ritchie and Spencer, 1994). Framework Analysis facilitates exploration of specific issues, while allowing for discovery of new and unexpected aspects of participants’ experiences or the way they assign meaning to phenomena (Gale et al., 2012). The method involves a systematic search for patterns via constant comparison of different groups, across different time points (e.g. young people, parents, health professionals, and volunteers; different categories of wishes; and different stages of wish process). Data analysis followed key steps of the framework approach: data familiarisation through repeated reading; coding of transcripts by assigning labels to salient pieces of data; developing a thematic coding framework; applying coding framework to whole data set (indexing); comparing within participant accounts and between participant groups; and data interpretation. Data storage, coding, and retrieval were supported by NVivo v12 (Lumivero). All participants were provided pseudonyms.

### Trustworthiness

To ensure rigour, data were independently coded by first and second authors alongside regular meetings with the third author, to compare and refine codes and then to dialogically generate descriptive followed by interpretative themes.

### Reflexivity

Our research team were white British, female researchers, trained in health psychology to post-doctoral level. Two researchers (GH and RK) are Registered Practitioner Psychologists and all work in the area of child health.

## Findings

### Participants

Fifty interviews were conducted, covering parents, children and young people, health professionals, and charity volunteers from a wide, UK-based geographical area. Twenty-two families were recruited and interviewed (23 parents, 17 young people). This included seven pre-wish families; six post-wish (≤8 weeks) families; and nine post-wish (≥8 weeks) families. Children varied in age (3-18 years); by health condition (e.g. congenital heart disease, leukaemia, and muscular dystrophy); and by wish type (e.g. to go, to have, and to meet). Five healthcare professionals were interviewed, including social workers, nurses, and a doctor. Seven charity volunteers were interviewed; volunteer experience ranged from 2 to 30+ years (Table 1).

**Table 1.** Participant characteristics.

**Families**				
**Parent**	**Child**	**Wish-child age at wish granting**	**Wish type**	**Wish-child health condition**
**Post-wish ≥8 weeks**				
Emma and Tom	Child not interviewed	14 years	To have	Cerebral palsy
Ava	Matthew	18 years	To go	Muscular dystrophy
Emily	Ellie	8 years	To meet	Cardiology
Beverley	Liam	4 years	To go	Respiratory
Parent not interviewed	Olivia	16 years	To go	Oncology
Carla	Peter	11 years	To meet	Oncology
Susan	Stuart	17 years	To go	Oncology
Mary	Ethan	6 years	To have	Oncology
Samira	Manni	4 years	To be	Oncology
**Post-wish ≤8 weeks**				
Donna	Michael	8 years	To go	Haematology
Paul	Child not interviewed	8 years	To go	Oncology
Anna	Amelia	10 years	To go	Congenital condition
Sarah	Summer	10 years	To meet	Genetic disorder
Amy	Erin	5 years	To go	Oncology
Lydia	Holly	10 years	To go	Respiratory
**Pre-wish**				
Lucy	Sophie	14 years	To go	Oncology
Katie	Child not interviewed	17 years	To meet	Genetic disorder
Julie	Child not interviewed	4 years	To go	Neurological
Zoe and Adam	Mason	8 years	To go	Cardiology
David	Eddie	14 years	To go	Haematology
Tracy	Child not interviewed	3 years	To go	Undiagnosed – neurological
Rachel	Jackie	18 years	To meet	Cerebral palsy
**Volunteers**				
**Participant**	**Volunteer role**	**Years in role**	**Years volunteering**	**Wishes facilitated**
Caroline	Wish visitor and fundraiser	6 years	6 years	100
Debbie	Wish visitor	30+ years	30+ years	400-500
Steve	Volunteer wish ambassador	2 + years	30+ years	400+
Jessica	Wish visitor	5 years	5 years	400
Michelle	Wish visitor	2.5 years	2.5 years	100
Claire	Wish visitor	10+ years	10+ years	200+
Amanda	Wish visitor and on celebrity team for wish attendance	7-8 years	7-8 years	100-150
**Health professionals**				
**Participant**	**Speciality and role**	**Years in role**	**Years referring to wish-granting charity**	**Referrals to wish-granting charity**
Wendy	Oncology – social worker	5	5	75
Karen	Oncology – community nurse			
Helen	Oncology and haematology –social worker	1	1	15
Faye	Paediatric oncology/haematology – social worker	8	8	90
Elaine	Rare diseases/renal – consultant	12	8	More than 50

Total = 23 parents; 17 young people; 7 volunteers; 5 health professionals.

### Themes

**Figure 1.**
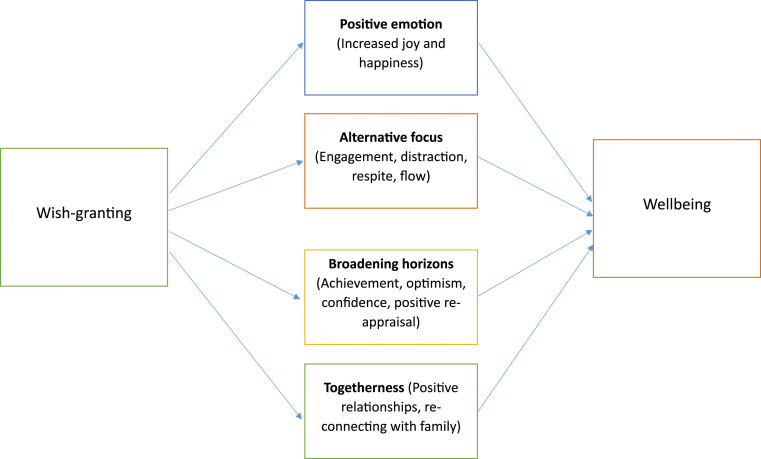
Mechanisms underpinning wish-granting interventions.

Analysis generated five core themes. Theme one describes the context in which wish interventions operate, *‘Shoved into a different world’*. This is important for understanding *how* wish interventions work: by increasing positive emotions *‘Everybody is just more happy’*; by broadening families’ horizons; by providing an alternative focus; and by fostering togetherness (Figure 1).

#### ‘Shoved into a different world’

Living with a child with a life-threatening health condition was a highly emotive and stressful experience, characterised by disruption and uncertainty. Navigating foreign territory upon diagnosis, families described alienation and grief stemming from their mourning of familiarity and lost futures. Accepting incurability of a child’s condition was punishing for families, who struggled to maintain structures and routines. Coping was an ongoing process, with new challenges presented daily.

For many families, their child’s condition meant prolonged stays in hospital and parents assuming a new role of ‘parent-carer’. While accepting this responsibility as a hugely important personal duty, parents were mindful of the demands and restrictions it placed on family life, including economic burden from reduced capacity to work and/or a need to privately fund carers or medical equipment. For many, being a ‘parent-carer’ was a relentless, full-time task where care planning became part of daily life. Yet, on accessing support, parents described guilt and worry about sharing this responsibility.

*‘The responsibility that you feel 24 hours a day, seven days a week is almost at times crushing, really. You can never relax. You always have to think two steps ahead of the day. You can’t do anything spontaneously, you can’t… everything has to be meticulously planned’. (Ava, Parent, Post-Wish ≥8 Weeks)*

#### ‘Everybody is just more happy’

On receiving news that their child’s wish would be granted, families described intense positive emotions of joy and excitement (often mixed with surprise and disbelief), particularly where wishes were perceived as a ‘*money can’t buy’* opportunity (*Sarah, Parent, Post-Wish ≤ 8 Weeks*). Children considered themselves ‘*lucky’* to have their wish granted and parents expressed extreme gratitude towards the wish-granting charity.

*‘I got off the phone and actually just burst into tears, it was very moving, yes. It was like, “Woah.” Yes, it was a big thing’. (Mary, Parent, Post-Wish ≥8 Weeks)*

*‘There’s no words really that you can use to express what it’s meant to us. I’ve repeatedly said, “Thank you, thank you, thank you.” You just don’t know how to express that, what it means because it might have a price for the airline ticket or a price for the hotel, a price for the Disney lunches but you can’t put a price on what they’ve given us’. (Lydia, Parent, Post-Wish ≤8 Weeks)*

Anticipation leading up to receiving a wish generated further excitement, with families reporting *‘living off adrenaline’ (Julie, Parent, Pre-Wish)*, comparing their excitement to a child on Christmas day *(Adam, Parent, Pre-Wish; Tracy, Parent, Pre-Wish)*. Parents observed beneficial effects of these positive emotions on their child’s emotional state, giving them *‘a massive boost’ (Olivia, Child, 16 years old, Post-Wish ≥ 8 Weeks)* and siblings also shared in their excitement. Supported by the wish-granting charity, families engaged in strategies to build anticipation (e.g. countdowns, family discussion, and token gifts). Fear of disappointment meant some families felt a need to control their child’s anticipation, by concealing wishes. While not distracting from the wish experience, it did mean that those children did not experience anticipatory excitement described by others.

On wish days, families felt they were given *‘VIP treatment’ (Amy, Parent, Post-Wish ≤ 8 Weeks)* with added extras such as limousine rides, favourite restaurant meals, spending money, and special passes. For some children, perceived rarity of their wish made them feel special: *‘it felt like I was a celebrity’ (Ellie, Child, 8 years old, Post-Wish ≥ 8 Weeks*), *‘treated like royalty’ (Donna, Parent, Post-Wish ≤ 8 Weeks)*. Wishes were frequently reported to exceed families’ expectations. Words such as *‘amazing’* (*Susan, Parent, Post-Wish ≥ 8 Weeks*), *‘magical’ (Sarah, Parent, Post-Wish ≤ 8 Weeks), ‘once in a lifetime’* (*Ava, Parent, Post-Wish ≥ 8 Weeks)*, and *‘incredible’* (*Paul, Parent, Post-Wish ≤ 8 Weeks)* were used by parents to describe wishes. For children, wishes were experienced as being *‘perfect’ (Matthew, Child, Post-Wish ≥ 8 Weeks*) *and ‘absolutely brilliant’ (Olivia, Child, 16 years old, Post-Wish ≥ 8 Weeks)*. Where children had limited communication, parents observed positive changes to their child’s body language, interpreted as positive emotion in response to their wish.

It was important to families that they maintained feelings of joy and happiness. They did so by engaging in strategies to keep wish memories alive. This included talking about wishes, looking through photographs and other mementos. Some families created special memory aids, for example, scrapbooks and photo frames. For younger children, mementos were incorporated into play to support reminiscence. Health professionals indicated that photos provided something tangible compared with ‘dream-like’ feelings of wishes.

*‘We’ve got a scrap book kit to be able to put together and we’ve got loads of photographs printed off… to be able to look back on with maps and receipts and a nice little memory book really. And sort of, for her to look through. Because I know she will. I know it’s something that she will keep up and look back on with fondness’. (Lydia Parent, Post-Wish ≤8 Weeks)*

#### Broadening horizons

For many families, living with a child with a life-threatening condition implied a shrinking lifeworld characterised by loss and isolation. Wishes that encouraged families to operate outside their *‘comfort zone’ (Ava, Parent, Post-Wish ≥ 8 Weeks)* were therefore perceived to unblock obstacles to families’ future orientation, providing glimpses of what could be achieved. For example, travel had been discounted by many families due to complexities in transporting their child and/or medical equipment. However, post-wish, families reported a shift in perspective, actively seeking opportunities for mobility rather than avoiding them. Wish experiences thus opened a world of future possibilities with families demonstrating strength and determination to engage with activities previously dismissed as unfeasible.

*‘It’s made us all feel a lot more confident about actually attempting to do these things. Being more adventurous with the kids rather than just do the same old holidays that we always do in the same old places. You know, the same old caravans and that sort of thing. It's made us all feel a lot more, well, we can you know, we can do it, we can go and have a nice time, we can look at the nicer side of a holiday rather than... I tend to book anything like this with, “What if this goes wrong, what if that goes wrong?” But I don't have that attitude now’. (Ava, Parent, Post-Wish ≥8 Weeks)*

Post-wish children also held a stronger sense of optimism for their future lives, broadening their perceptions of what might be possible. Parents and health professionals affirmed increased vitality in children, *‘a new, renewed energy’ (Elaine, Health professional)*. Upon returning from their wish, children reported feeling more confident and better able to cope with problems, often linked to a sense of achievement gained through their wish. Examples included a willingness and determination to engage with their treatment, healthcare appointments, and medical procedures.

*‘It’s shown me that he can do it, it’s shown me that he can deal with these things... It’s given him a sense of independence, a sense that he can do these things. That he doesn’t need me or his Dad there the whole time’. (Ava, Parent, Post-Wish ≥8 Weeks)*

Wishes also opened the world to children by altering their perceptions of self. Children reported more positive views of themselves and their health condition post-wish because wishes supported a view that positive things can result from having a life-limiting health condition.

*‘I think it has made me notice more the good things that can come out of the condition, things like that, and not just the fact that I’ve got to carry injections or take lots of medicines and things like that’. (Eddie, Child, 14 years old, Pre-Wish)*

Changes in self-perception were also associated with the presentation of wishes as recognition of what children had been through. Children found comfort in the idea that someone took note of how life was for them, recognising that their diagnosis was *‘really rubbish’ (Olivia, Child, 16 years old, Post-Wish ≥ 8 Weeks)*. They appreciated that their courage and determination had been noticed.

*But I know there was* *that little part of me that wanted to say, ‘Just because I’m saying thank you and just because I’m saying I’m fine, I’m not fine, please know I’m not fine.’ And, to me, that’s what [wish-granting charity] was acknowledging with me, ‘We know you weren’t fine and that’s why you’re getting this wish even though you kept a smile on your face’. (Olivia, Child, 16 years old, Post-Wish ≥8 Weeks)*

Following wishes, parents felt able to make positive changes, to improve their own wellbeing as well as reassessing their priorities and even careers. Being a wish recipient also led to feelings of increased benevolence for families, leading to a desire to raise awareness of their child’s health condition, as well as fundraise for the wish-granting charity to ‘give something back’.

*‘It just more or less changed our outlook on life that, you know, there’s more to life than working your fingers to the bone and not seeing each other. And it made us re-evaluate, that’s why I handed my notice in at work’. (Emily, Parent, Post-Wish ≥8 Weeks)*

In cases where it was possible for a child to enter remission, wishes provided families with a sense of closure, marking their first step on a road to recovery: *‘putting a full stop to his illness… end[ing] that hurt on a high note’ (Paul, Parent, Post-Wish ≤ 8 Weeks)*.

#### An alternative focus

During difficult times, wishes provided something to look forward to or a short respite from the challenges of daily life. ‘To Go’ wishes, in particular, provided respite from routine medical care and worry that accompanied it, facilitating a sense of ‘normality’ for families who felt able to experience a world in which ‘life’ was prioritised over illness. This alternative focus, even if only for a brief time, provided an opportunity to *‘escape’* daily stresses and strains *(Anna, Parent, Post-Wish ≤ 8 Weeks)*.

Healthcare professionals and parents used wishes to distract children from their illness and treatment, particularly where a child’s health condition required medical intervention. Pre-wish, parents constructed wishes as *‘a light at the end of the tunnel’ (Lucy, Parent, Pre-Wish; Olivia, Child, 16 years, Post-Wish ≥ 8 Weeks; Paul, Parent, Post-Wish ≤ 8 Weeks)*. Professionals reiterated that having *‘something to look forward to’ (Faye, Health Care Professional)* motivated children through treatment. When a child required further medical procedures following their wish, families used positive memories to support them.

*‘Every day he struggles having his feed because he has to be attached to a feeding pump for a couple of hours every day. And knowing that he has got the wish and he has got Disney to look forward to, you can distract him for some period of the time. It doesn’t always work but for the majority it does, but it really helps when you have to turn the tube because he hates it, so it really really helps in that way’. (Zoe, Parent, Pre-Wish)*

Upon returning from their wish, health professionals and parents described cases in which children had been more determined and engaged with their treatment and felt more positive about taking their medication. This newfound positive outlook resulted in children being more likely and more confident to adhere to their treatment regimen, improving health outcomes.

*‘The times when it’s most strong, is when it seems the most difficult and it seems the darkest, its then I find, it’s then it’s [the wish] got a really big impact. And sometimes it can lift them out of that horrible place that they are at the time and think about something else, which is good’. (Elaine, Health Professional)*

Where parents believed their child was not well enough to receive a wish, they often requested it be delayed. However, delaying a wish did not positively impact all children. For example, receiving a wish after gaining ‘closure’ from their illness was not associated with the kinds of positive impacts that children who were still undergoing treatment reported. Wish timing was therefore constructed as important for generating impact. Wishes often represented a first for parents in relinquishing responsibility for any aspects of their child’s care. Parents appreciated being involved in wish organisation, building trust in the process, and feeling able to relax and enjoy the lead up to their child’s wish.

#### Fostering togetherness

Wishes that facilitated quality time together were highly valued and conceived as benefiting whole families. Examples included external activities such as holidays and days out and also those within the home such as a waterbed located in the heart of the home which enabled a child to participate in daily family life.

Being together and experiencing together was particularly important for reconnecting as a family. Healthcare professionals described this in terms of families being able to reconstruct their family story; doing so was not only important for families celebrating the end of their child’s treatment but also for families whose child’s health condition was ongoing or terminal.

Being together was also viewed as important for siblings, who were often perceived as overlooked. Wishes were reported to positively enhance relationships between children and their sibling. When siblings were included in wishes, parents reported they were treated similarly to the wish child, allowing siblings to also experience wish magic.

*‘I think people, families, kind of need that constant, sort of, bringing together and, you know, reapplying the social glue that you have, I guess. Because when you’ve got a medical condition, it can be stretched somewhat, you know. Parents have arguments, whatever and you, kind of, that you might not have in a normal family situation. But it’s, kind of, hopefully going to be a, yes, a reapplication of those social bonds between the family that help us stick together. Plus, you know, it’s another thing to talk about that’s not hospital appointments and medical things, you know. Lots of the discussion is about those kinds of things and it’s nice to have something else to, you know, discuss and have a sort of shared part in a family story or narrative that’s not about that. So, I think that’s all going to be positive’. (David, Parent, Pre-Wish)*

Wishes were also found to be crucial in providing parents and partners with much-needed quality time together. While parents often faced spending a large amount of time apart, health professionals described witnessing how this could damage relationships. Parents who felt wishes had brought them closer together as a couple, repairing and restoring their relationships and allowing them to share quality time together, reported receiving a greater wish impact.

## Discussion

This study sought to explore families’ experiences of receiving wish-granting interventions to understand how wishes impact wellbeing. Findings suggest wishes improve family wellbeing by increasing positive emotions, broadening families’ horizons, providing an alternative focus, and fostering togetherness. While previous research found wish-granting to improve health behaviours and outcomes for children and families (Heath et al., 2021), this study adds to our understanding of *how* wishes bring about these benefits. Such understanding is crucial to maximising the impact of wishes for future recipients.

Consistent with existing literature, families in our study described significant impacts of living with a child with complex healthcare needs (Bally et al., 2018; Fraser et al., 2021). For all recipients, however, wishes generated positive emotions of joy and happiness, supporting research that wish-granting improves wellbeing (Chaves et al., 2016; Darlington et al., 2013; Shilling and Sarigiani, 2014; Shoshani et al., 2016). Positive emotion is a key component in wellbeing theory (PERMA) (Seligman, 2012), and paediatric interventions aiming to increase positive emotion (e.g. clown doctors, play therapy) are well-established as improving outcomes for hospitalised children (Godino-Ianez et al., 2020; Lopes-Júnior et al., 2020).

While previous research suggests positive emotions may not be maintained post-wish (Darlington et al., 2013), families in our study described enduring benefits with some reporting they still experienced emotional benefit from wishes over a decade later. As with other studies (e.g. Schilling and Sarigiani, 2014), families preserved and revived happy wish experiences through use of aide-memoire (e.g. journals, scrapbooks, and photographs), particularly during difficult times. Maintaining positive effects of wishes in this way can be understood as a form of savouring, an act of consciously capturing a *present* experience for subsequent reflection (Biskas et al., 2019). Research suggests savouring interventions can be effective for increasing intensity and duration of positive emotion, in both young people and adults (Smith and Hollinger-Smith, 2014; Villani et al., 2023).

Like previous research (Deschenes, 2009), our study highlights the importance of anticipation/reminiscence in distracting children from medical procedures and in boosting morale. By shifting a child’s focus to something engaging and absorbing, distraction has been shown a promising intervention for children, demonstrating improved capacity to manage pain and anxiety-provoking experiences (Bukola and Paula, 2017; Koller and Goldman, 2012). In addition, engagement with a child’s wish experience was shown to offer respite from daily worries. Such a state is seen in experiences of ‘flow’ (Seligman and Csikszentmihalyi, 2000), which have previously been shown to contribute to wellbeing.

Our study demonstrated wishes as broadening families’ perspectives of what might be possible going forward, spatially, relationally, and temporally. This boosted confidence and self-esteem, encouraging family members to view themselves and their child’s health condition more positively, including views that good things could result from living with illness. Receiving a wish thus facilitated positive reappraisal of future possibilities. Considered a form of meaning-based coping, positive reappraisal has previously been shown to act as a key resilience mechanism, leading to increased wellbeing (Riepenhausen et al., 2022). Parents also described changes in their priorities following wishes, focusing more on purpose, values, and strengths. Wishes may then be seen as a way of supporting families to transform meaning from suffering related to their child’s condition and treatment, offering a ‘catalyst’ for positive change or pathway for post-traumatic growth (Emmons, 2003).

Finally, wishes were perceived to facilitate opportunities for family togetherness, reducing feelings of isolation and promoting re-connection and re-construction of a family’s story. Positive relationships are a cornerstone of wellbeing in young people (Blum et al., 2022), consistently shown to determine wellbeing, even in challenging circumstances (Ryan and Deci, 2000; Seligman, 2012).

### Strengths and limitations

As the first UK study of its kind, findings contribute to our understanding of how wish-granting interventions impact wellbeing for recipients. Our study includes a large sample that is varied in age, health condition, and wish type. It thus broadens previous research that focuses on children with cancer and allows for contrasting experiences to be incorporated. However, the study does have limitations. First, extremely ill children and those who lacked language or cognitive abilities to participate in interviews (on their own or with support) were not included, nor were siblings or those families where a child had died following wish fulfilment. We therefore lack first-hand accounts of how wishes impacted these individuals. In addition, parents consisted primarily of mothers. While reflective of the care-giver role more generally (Smith et al., 2015), father’s experiences remain underrepresented. Families who had a positive wish-granting experience might also have been more likely to participate, meaning we may have missed those whose experiences were less positive. Finally, while providing an in-depth, experiential account of wish-granting and wellbeing, causal relationships cannot be inferred using a qualitative approach.

### Future research

Further research is needed to understand the impact of wishes by wish type and by condition, to explore if certain wish types (e.g. To Go wishes) have a bigger impact, considering the importance of broadening horizons. With our (and others’) studies suggesting that wish impacts extend to other family members (e.g. siblings and grandparents), future research is needed to understand their experiences directly. Further research is also needed to examine the impact of wishes on parental bereavement outcomes. Finally, mechanisms underpinning wish-granting interventions in this qualitative study could be examined quantitatively, including outcomes on healthcare utilisation, health behaviours (e.g. treatment adherence), and standardised measures of wellbeing.

### Implications for practice

Based on our study, several recommendations for increasing and maintaining wish impact can be made. First, strategies designed to increase anticipation and keep wish memories alive may increase duration and intensity of positive emotion. Such strategies could include count-down calendars, collages or drawings about the child’s hopes for their wish, creating memory boxes, keeping diaries and scrap books, and encouraging children (and siblings) to talk about their wish. Second, supporting young people to choose wishes that take them beyond their perceived limitations may foster a sense of personal achievement and change in perceptions of future possibilities (positive reappraisal). Third, facilitating social connections with other wish families via post-wish events would provide opportunities for families to share their stories with other families of children with critical illness.

## Conclusion

This study shows wish-granting interventions foster wellbeing for children with life-threatening health conditions, by increasing positive emotions, by broadening families’ horizons, by providing an alternative focus, and by providing opportunities for togetherness. Recommendations to grow and maintain impact include developing strategies to increase anticipation and keep wish memories alive (savouring); encouraging children to consider wishes that take them beyond their perceived limitations; and facilitating families to share their experiences and ‘give back’ to the community.
